# New machine-learning models outperform conventional risk assessment tools in Gastrointestinal bleeding

**DOI:** 10.1038/s41598-025-90986-1

**Published:** 2025-02-21

**Authors:** Eszter Boros, József Pintér, Roland Molontay, Kristóf Gergely Prószéky, Nóra Vörhendi, Orsolya Anna Simon, Brigitta Teutsch, Dániel Pálinkás, Levente Frim, Edina Tari, Endre Botond Gagyi, Imre Szabó, Roland Hágendorn, Áron Vincze, Ferenc Izbéki, Zsolt Abonyi-Tóth, Andrea Szentesi, Vivien Vass, Péter Hegyi, Bálint Erőss

**Affiliations:** 1 Institute for Translational Medicine, Medical School, University of Pécs, Pécs, Hungary; 2 Department of Stochastics, Institute of Mathematics, Budapest University of Technology and Economics, Budapest, Hungary; 3 Centre for Translational Medicine, Semmelweis University, Budapest, Hungary; 4 Institute of Pancreatic Diseases, Semmelweis University, Budapest, Hungry Hungary; 5 Fejér County Szent György University Teaching Hospital, Székesfehérvár, Hungary; 6 First Department of Medicine, Medical School, University of Pécs, Pécs, Hungary; 7 Institute of Biostatistics and Network Science, Semmelweis University, Budapest, Hungary; 8 Department of Biostatistics, University of Veterinary Medicine, Budapest, Hungary; 9 Internal Medicine, Hospital and Clinics of Siófok, Siófok, Hungary; 10 Department of Gastroenterology, Central Hospital of Northern Pest – Military Hospital, Budapest, Hungary; 11 Selye János Doctoral College for Advanced Studies, Semmelweis University, Budapest, Hungary

**Keywords:** Gastrointestinal bleeding, Machine learning

## Abstract

Rapid and accurate identification of high-risk acute gastrointestinal bleeding (GIB) patients is essential. We developed two machine-learning (ML) models to calculate the risk of in-hospital mortality in patients admitted due to overt GIB. We analyzed the prospective, multicenter Hungarian GIB Registry’s data. The predictive performance of XGBoost and CatBoost machine-learning algorithms with the Glasgow-Blatchford (GBS), pre-endoscopic Rockall and ABC scores were compared. We evaluated our models using five-fold cross-validation, and performance was measured by area under receiver operating characteristic curve (AUC) analysis with 95% confidence intervals (CI). Overall, we included 1,021 patients in the analysis. In-hospital death occurred in 108 cases. The XGBoost and the CatBoost model identified patients who died with an AUC of 0.84 (CI:0.76–0.90; 0.77–0.90; respectively) in the internal validation set, whereas the GBS and pre-endoscopic Rockall clinical scoring system’s performance was significantly lower, AUC values of 0.68 (CI:0.62–0.74) and 0.62 (CI:0.56–0.67), respectively. ABC score had an AUC of 0.77 (CI:0.71–0.83). The XGBoost model had a specificity of 0.96 (CI:0.92–0.98) at a sensitivity of 0.25 (CI:0.10–0.43) compared with the CatBoost model, which had a specificity of 0.74 (CI:0.66–0.83) at a sensitivity of 0.78 (CI:0.57–0.95). XGBoost and the CatBoost models evaluate the mortality risk of acute GI bleeding better, than the conventional risk assessment tools.

**Supplementary Information:**

The online version contains supplementary material available at 10.1038/s41598-025-90986-1.

## Introduction

Despite the changes in epidemiology and management of acute gastrointestinal bleeding (GIB) in the last three decades, the mortality is still high (2–20%)^1^.In a large Danish upper GIB (UGIB) cohort of 12,601 patients, the mortality of haemodynamically unstable patients was 13%, whereas it was 3.8% in the haemodynamically stable group^2^. In a prospective French UGIB cohort, the mortality was 16.8% in the in-patient and 5.8% in the out-patient group^3^. In a recent systematic review, using data from 41 studies, the case-fatality rate ranged from 0.7 to 4.8% for UGIB and 0.5–8.0% for lower GIB (LGIB)^4^.

Careful risk assessment of patients in the emergency care unit to identify high-risk patients early can be a potential solution to minimize the mortality of GIB. High-mortality risk patients might need admission to the intensive care unit (ICU), require more transfusion, fluid resuscitation, vasopressors, and even have a higher need for endoscopic intervention^5,6^.

Many risk assessment tools, such as Glasgow-Blatchford score (GBS)^6^, pre-endoscopic Rockall score^7^, AIMS65^8^, PNED^9^, full Rockall score^10^, T-score^11^, and MAP(ASH)^12^, were developed to assess the risk of various outcomes in UGIB patients. ABC score is good for predicting mortality in both UGIB and LGIB^13^. A comparison of these conventional risk scoring systems suggested that GBS is reliable in selecting low-risk patients for out-patient management, although the accuracy of predicting mortality, rebleeding, and need for endoscopic treatment was relatively low^14^. Other analyses proposed that different risk scores perform better for elderly and younger patients^15^. The clinical use of risk scoring systems was criticized due to these controversies^16^.

The application of artificial intelligence to medicine has made substantial progress in the last decade because of the necessity to handle the vast amount of available clinical data effectively^17,18^.

As a type of artificial intelligence, a machine-learning (ML) algorithm builds a model based on a training dataset and can improve its performance with experience. ML is anticipated to be a tool for predicting individualized diagnoses and clinical outcomes, as it is more accurate and precise than traditional statistical analyses^18^. ML is ideal for analyzing large, complex, heterogeneous, and imbalanced datasets^1^.

The Hungarian Registry of Acute GIB was established to collect comprehensive data on patients and follow up on their hospital management. In this study, we aimed to develop and validate ML models to calculate the risk of in-hospital mortality in patients admitted for overt GIB, which can help triage suspected GIB patients, regardless of the bleeding source, into high- and low-risk mortality groups.

## Results

### Basic characteristics of the cohort

A total of 1,021 patients were included; the median age was 70 years (IQR:61–80); 60% were men. According to bleeding source, 527 patients (52%) had nonvariceal UGIB, 91 (8.9%) had variceal bleeding, 303 (30%) had LGIB, 23 (2.3%) had small bowel bleeding, and in 77 cases (7.5%) the bleeding source was iatrogenic. GIB was the reason for hospitalization in 82% of the cases (out-patients), and in 18% of the cases, GIB started in already hospitalized individuals (in-patients). In-hospital mortality was 11% in our cohort (108 patients). Detailed characteristics of the cohort are in Table 1.

**Table 1:** Basic characteristics of the Hungarian GIB cohort.

Characteristics	Mean (SD)	Median (IQR)	Missing, *n* (%)
Age (years)	69.5 (13.6)	70 (61–80)	0
Male sex (n, %)	611 (60%)		0
	**Yes**,** n(%)**	**No**,** n (%)**	**Missing**,** n (%)**
Smoking	198 (19%)	632 (62%)	191 (19%)
Regular alcohol consumption	205 (20%)	642 (63%)	174 (17%)
Haemodynamic instability on admission	160 (15.7%)	813 (79.6%)	48 (4.7%)
Melaena	421 (41.2%)	503 (49.3%)	97 (9.5%)
Haematochezia	392 (38.4%)	491 (48.1%)	138 (13.5%)
Gastroscopy as the first endoscopy	735 (81.8%)	163 (18.2%)	123 - no endoscopy
Intervention at first endoscopy	280 (31.2%)	618 (68.8%)	123 - no endoscopy
**Laboratory results**	**Mean (SD)**	**Median (IQR)**	**Missing**,** n (%)**
Haemoglobin (g/L)	96.0 (30.8)	95 (73–119)	77 (7.5%)
Platelet (G/L)	277 (147.6)	254 (185–343)	80 (7.8%)
CRP (mg/L)	33.6 (58.9)	10.3 (2.9–36.7)	156 (15.3%)
Creatinine (µmol/L)	120.7 (96.3)	93.00 (71–128.8)	143 (14%)
INR	1.7 (2.1)	1.2 (1.1–1.5)	218 (21.4%)
Systolic blood pressure (Hgmm)	121.6 (27.8)	120 (100–140)	87 (8.5%)
**Scores**	**Mean (SD)**	**Median (IQR)**	**Missing**,** n (%)**
Glasgow-Blatchford score	9.2 (4.6)	10 (6–13)	176 (17.2%)
Pre-endoscopic Rockall score	4.1 (1.5)	4 (3–5)	44 (4.3%)
Glasgow Coma Scale	13–15 points: 825 (80.8%)	9–12 points: 11 (1.1%)	179 (17.5%)
	<=8 points: 6 (0.59%)		
**Medications**	**Yes**,** n (%)**	**No**,** n (%)**	**Missing**,** n (%)**
Aspirin	207 (20.3%)	809 (79.2%)	5 (0.5%)
Clopidogrel	127 (12.4%)	889 (87.1%)	5 (0.5%)
LMWH	91 (8.9%)	925 (90.6%)	5 (0.5%)
DOAC	133 (13%)	883 (86.5%)	5 (0.5%)
Coumarin	105(10.3%)	911 (89.2%)	5 (0.5%)
NSAIDs	148 (14.5%)	868 (85%)	5 (0.5%)
**Co-morbidities**	**Yes**,** n (%)**	**No**,** n (%)**	**Missing**,** n (%)**
Liver disease	217 (21.3%)	804 (78.7%)	0
Thromboembolic diseases	117 (11.5%)	903 (88.4%)	1 (0.1%)
Heart failure	137 (13.4%)	884 (86.6%)	0
Atrial fibrillation or flutter	236 (23.1%)	785 (76.9%)	0
Diabetes mellitus	294 (28.8%)	727 (71.2%)	0
Chronic kidney disease	336 (32.9%)	618 (60.5%)	67 (6.6%)
Previous GIB	328 (32.1%)	693 (67.9%)	0

DOAC: direct oral anticoagulant, GIB: gastrointestinal bleeding, INR: international normalized ratio, IQR: interquartile range, LMWH: low-molecular-weight heparin, NSAID: nonsteroidal anti-inflammatory drug, SD: standard deviation, CRP: C-reactive protein.

### Evaluation of the machine-learning models

**Fig. 1:**
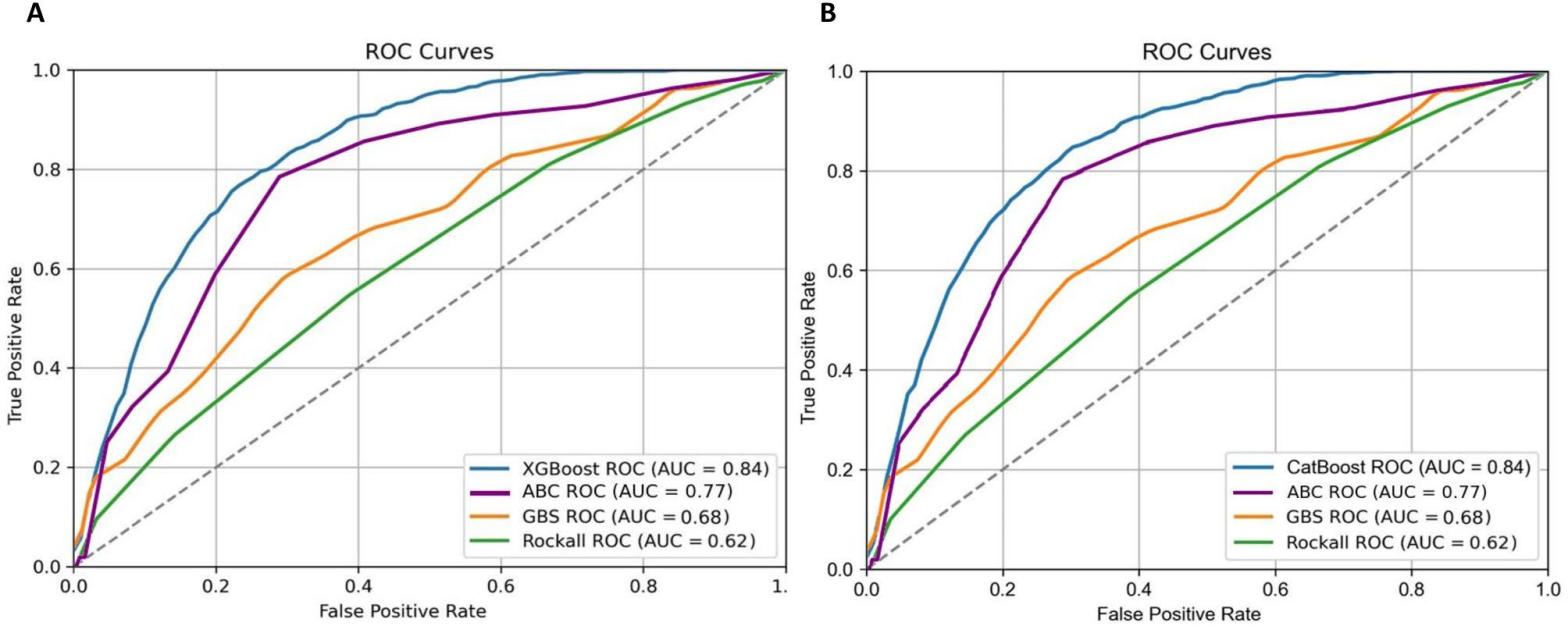
ROC curves of the machine-learning models compared with the performance of Glasgow-Blatchford, pre-endoscopic Rockall and ABC scoring systems. Figure 1(**A**) represents the XGBoost model. Figure 1(**B**) represents CatBoost model. AUC: area under the receiver operating characteristic curve, GBS: Glasgow-Blatchford score, ROC: receiver operating characteristic.

The XGboost and the CatBoost model identified patients who died with an AUC of 0.84 (CI: 0.76–0.90; 0.77–0.90; respectively) in the internal validation set, whereas the GBS and pre-endoscopic Rockall clinical scoring system’s performance was significantly lower, AUC values of 0.68 (CI: 0.62–0.74) and 0.62 (CI: 0.56–0.67) respectively (Fig. 1). ABC score hade an AUC of 0.77 (0.71–0.83) (Fig. 1).

**Fig. 2:**
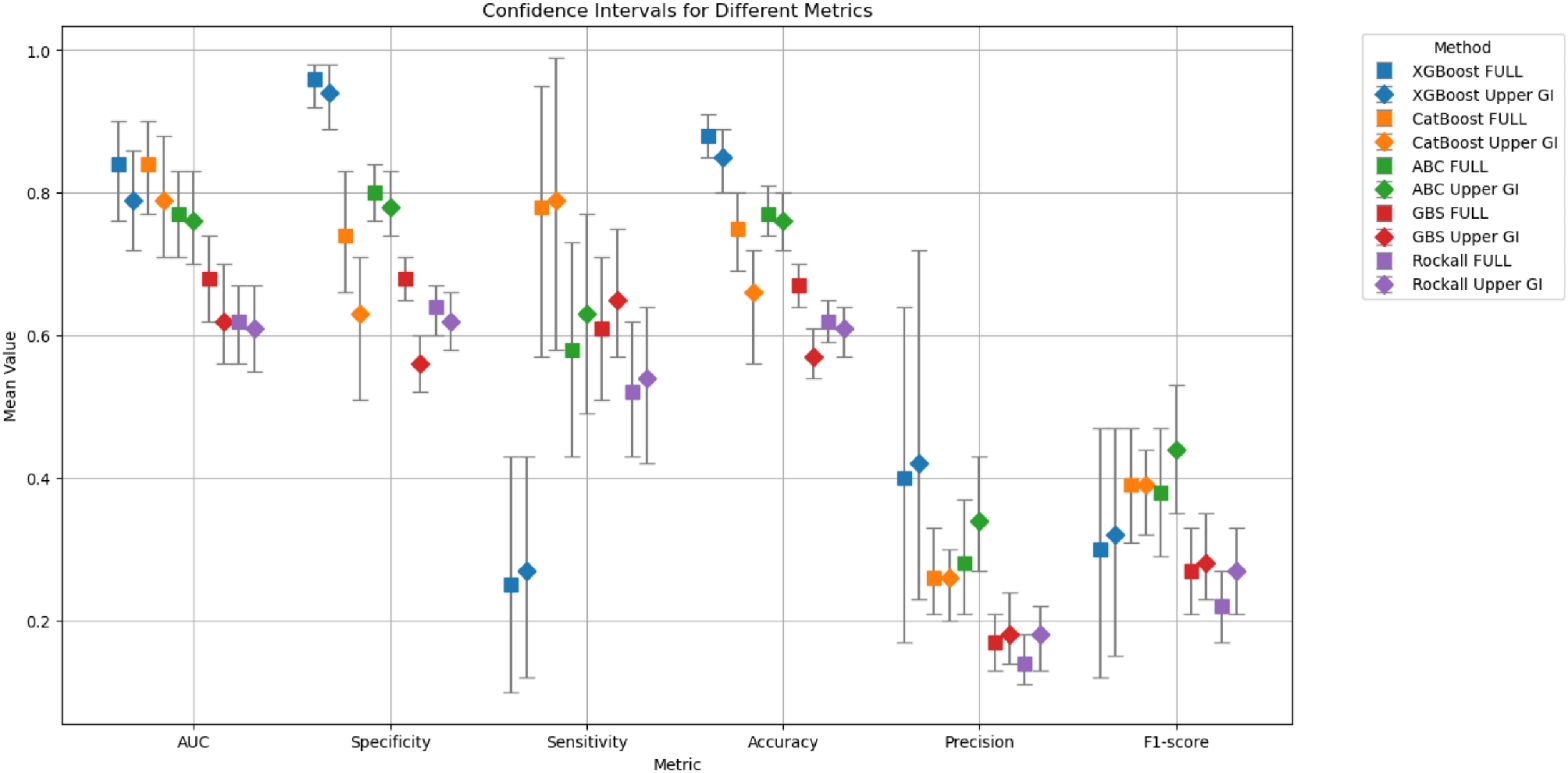
Comparison of XGBoost and CatBoost models with conventional risk assessment scores. Squares are representing the values of the total cohort, diamonds are representing the values in the upper GI bleeding subgroup.

We compared the models’ specificity, sensitivity, accuracy, precision, and F1 score (Fig. 2, Supplementary Table 1). The XGBoost model had an accuracy of 0.88 (CI: 0.85–0.91) and a sensitivity of 0.25 (CI: 0.09–0.43) compared with the CatBoost model, which had an accuracy of 0.75 (CI: 0.69–0.80) and a sensitivity of 0.78 (CI: 0.57–0.95). The specificity of the two models was 0.96 (CI: 0.92–0.98) and 0.74 (CI: 0.66–0.83), respectively. The XGBoost model shows high specificity but low sensitivity, limiting its utility for identifying high-mortality-risk patients. Conversely, CatBoost has a better sensitivity, so it can better identify low-mortality risk patients.

Metrics of the ABC, GBS and pre-endoscopic Rockall scoring systems were calculated (Supplementary Table 1); sensitivity was 0.58 (CI: 0.43–0.73); 0.61 (CI: 0.51–0.71) and 0.52 (CI: 0.43–0.62), respectively.

### Subgroup analyses of upper GI bleeding patients

In case of upper GI bleeding, the XGBoost and the CatBoost model identified patients who died with an AUC of 0.79 (CI: 0.72–0.86; 0.71–0.88; respectively) The GBS and pre-endoscopic Rockall clinical scoring system’s performance was significantly lower, AUC values of 0.62 (CI: 0.56–0.70) and 0.61 (CI: 0.55–0.67) (Fig. 2, Supplementary Table 1, Supplementary Fig. 1). The AUC value of the ABC score in this subgroup of patients was 0.76 (CI: 0.70–0.83) (Supplementary Fig. 1).

The XGBoost model had a sensitivity of 0.27 (CI: 0.12–0.43) compared with the CatBoost model, which had a sensitivity of 0.79 (CI: 0.58–0.99). The specificity of the two models was 0.94 (CI: 0.89–0.98) and 0.63 (CI: 0.51–0.71), respectively. Metrics of the ABC, GBS and pre-endoscopic Rockall scoring systems were calculated (Supplementary Table 1); sensitivity was 0.63 (CI: 0.49–0.77); 0.65 (CI: 0.57–0.75) and 0.54 (CI: 0.42–0.64), respectively.

### Interpretation of the machine-learning prediction models

**Fig. 3:**
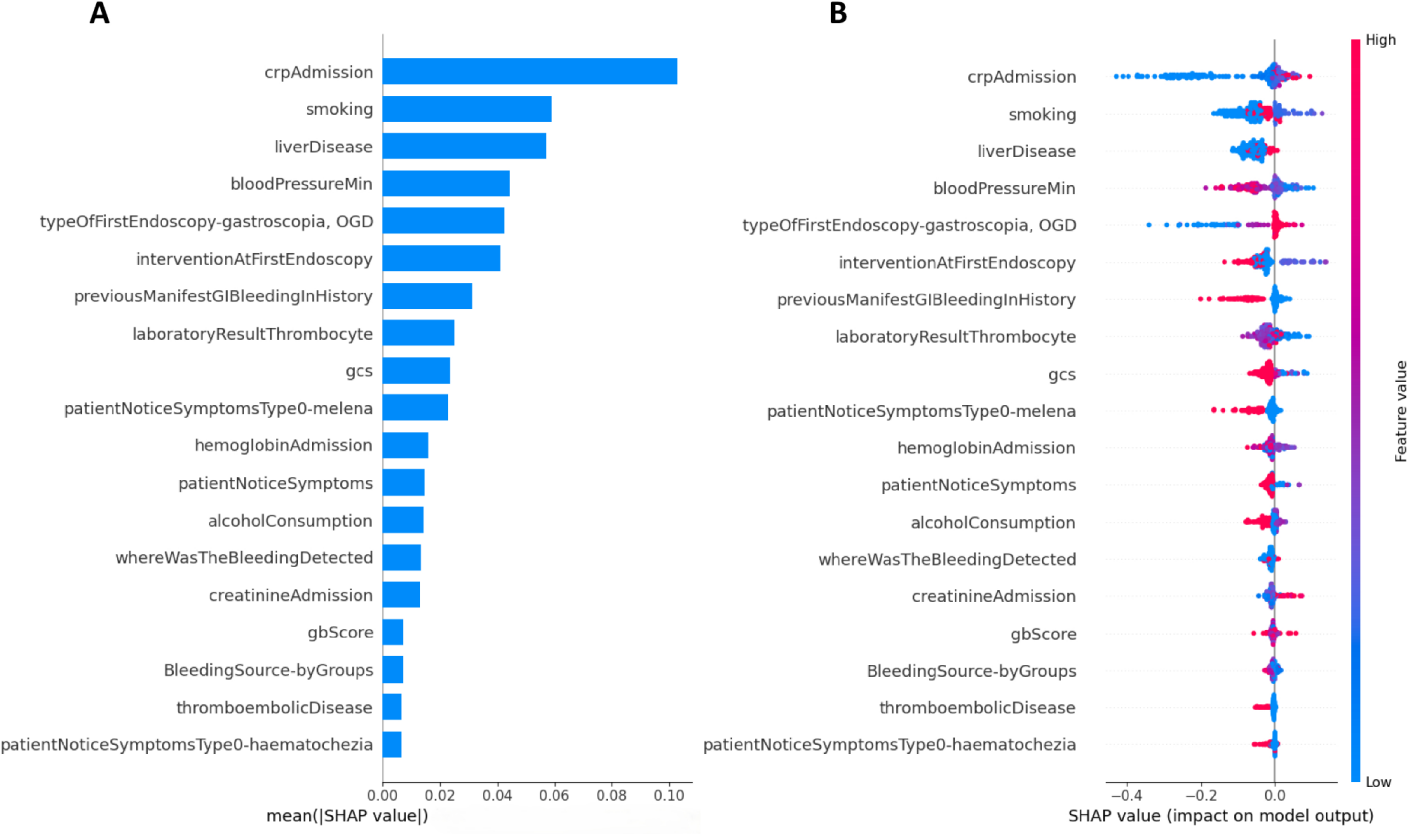
Summary SHAP plot of the impact of the features on the prediction of the XGBoost model. Figure 3(**A**) represents the mean absolute values of the feature’s SHAP values. In Fig. 3(**B**), each patient is visualized with a point on the beeswarm plot. A positive SHAP value indicates that the feature value contributes positively to the mortality risk. gbScore: Glasgow-Blatchford score, gcs: Glasgow Coma Scale, OGD: oesophagogastroduodenoscopy.

**Fig. 4:**
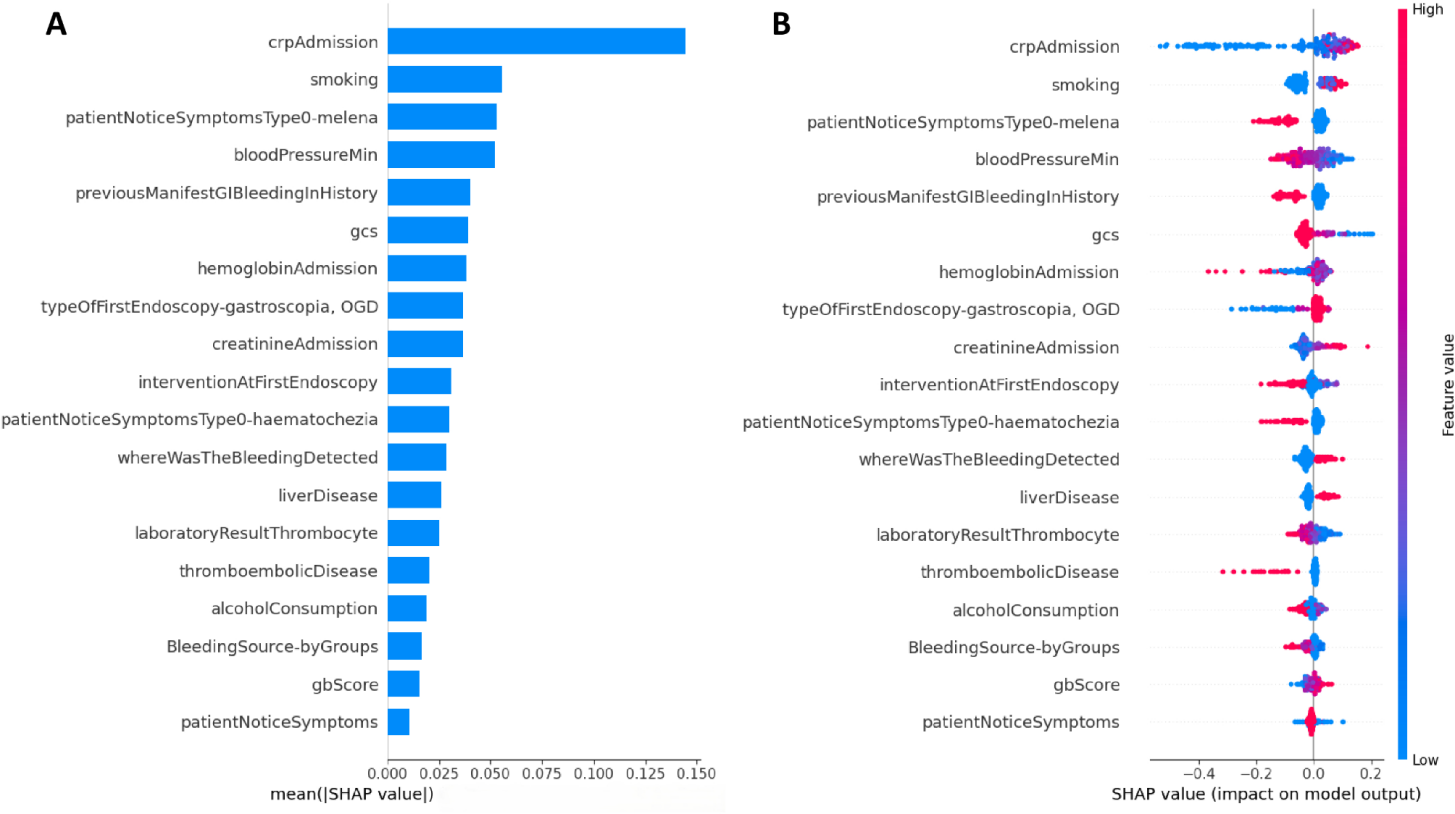
Summary SHAP plot of the impact of the features on the prediction of the CatBoost model. Figure 4(**A**) represents the mean absolute values of the feature’s SHAP values. In Fig. 4(**B**), each patient is visualized with a point on the beeswarm plot. A positive SHAP value indicates that the feature value contributes positively to the mortality risk. gbScore: Glasgow-Blatchford score, gcs: Glasgow Coma Scale, OGD: oesophagogastroduodenoscopy.

To explain our risk assessment models, we employed the SHapley Additive exPlanations (SHAP) method. The features involved in the models are listed in descending order according to their influence on the prediction (Figs. 3A and 4A). The seven most important elements of the XGBoost model were CRP level, smoking, liver disease, minimum systolic blood pressure, gastroscopy as the first endoscopy, intervention at first endoscopy, and previous GIB; in the CatBoost model, the most influential conditions were CRP level, smoking, melaena, minimum systolic blood pressure, previous GIB, Glasgow Coma Scale (GCS) and haemoglobin level.

In Figs. 3B and 4B, the SHAP value of every feature in every case is visualized with a point on a summary plot. A positive SHAP value indicates that the feature value contributes positively to the mortality risk, while a negative SHAP value means that the feature value decreases the predicted mortality risk.

High CRP levels on admission, low platelet count, low haemoglobin level, low systolic blood pressure, high creatinine level at admission, and low GCS score increased mortality risk. Some features can be interpreted as protective factors, such as no smoking, lack of liver disease, not gastroscopy as the first endoscopy, melaena noticed by the patient, known previous GIB episode, and presentation as an out-patient.

**Fig. 5:**
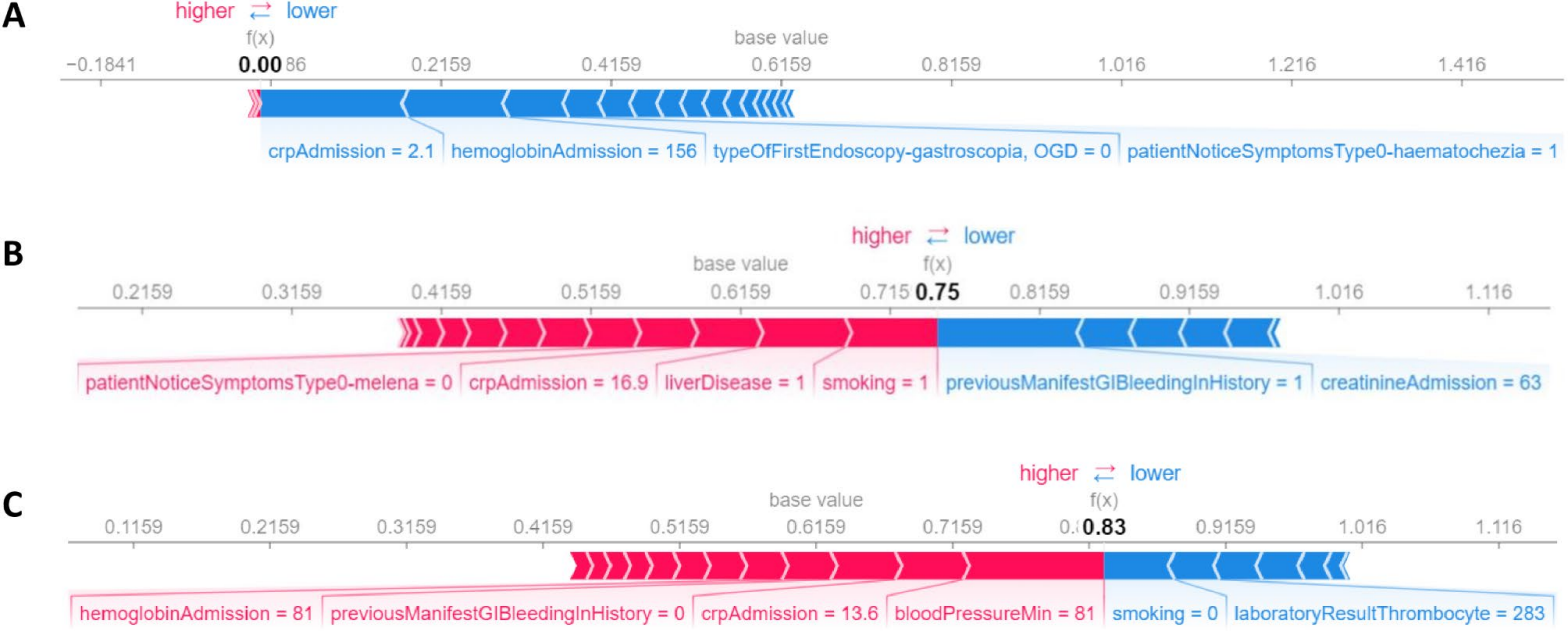
Interpretation of risk assessment using the SHAP method. Figure 5(**A**, **B**, and **C**) represent the risk assessment of different individuals.

In Fig. 5, three different cases are shown to explain how our CatBoost model calculated the mortality risk of these patients. The red bars represent the characteristics that converge towards a higher probability of death; the blue bars represent the characteristics that lower the mortality risk. The length of the bars is proportional to the influence of the feature in the prediction. In the first patient’s case (Fig. 5A), the model predicted 0 mortality risk because he had favorable features according to the risk assessment model. The second patient (Fig. 5B) had a 0.75 probability of mortality primarily due to liver disease and smoking; on the risk-lowering side of the prediction, the patient had a normal creatinine level on admission and had a previous GIB episode. In the third case (Fig. 5C), the model assessed the highest mortality risk mainly because of the low minimum systolic blood pressure, slightly elevated CRP, low haemoglobin level, and no previous known GIB. We can also establish the protective role of no smoking and normal platelet count.

## Discussion

We developed two ML-based mortality risk assessment tools feasible in acute GIB and compared their performances to GBS’s, pre-endoscopic Rockall score’s and ABC score’s performance. Our study is a multicenter, observational study with prospective and retrospective data collection involving data from 1,021 patients. The mortality risk of each patient can be calculated and the value of the score is between 0 and 1. The performance was measured in AUC to evaluate our ML-based risk assessment tools. The AUC of the XGBoost and CatBoost models are both 0.84, which is considered a good performance, whereas GBS and pre-endoscopic Rockall scores had significantly lower AUC. The performance of our ML-based models was better than the performance of ABC score (AUC:0.77).

We analyzed six metrics of both ML models and found that the CatBoost model had a significantly higher sensitivity. The specificity was significantly higher in the XGBoost model, which means it finds the true negative patients better but has a low sensitivity of 0.25, so it can’t reliably detect GI bleeding patients with mortality risk, especially if the mortality risk is low. In GIB-related mortality risk stratification, it is essential to have a model with good sensitivity to avoid any untoward outcome. For a test to be useful, sensitivity + specificity should be at least 1.5 according to the explanation of Power and Fell^19^. Based on that, we recommend using the CatBoost model in decision-making, which has good sensitivity (0.78) and specificity (0.74). Thus, the CatBoost model with the current optimalizations is better in ruling out death and is better in identifying even low mortality risk patients, than the XGBoost model.

During development, we did not differentiate patients according to their bleeding source, and we consider that to be one of the most unique qualities of our study. Hence, the risk assessment tool is designed to be applied regardless of the suspected source of bleeding, which promotes a universal use of our CatBoost model in case of acute GIB. When we examined the performance of XGBoost and CatBoost models in the upper GI bleeding subset of patients, there was no significant difference in the upper GI subgroup (AUC:0.79) and in the total GI bleeding cohort (AUC:0.84). We assume, the underlying reason for the slightly higher performance in the total cohort is that the ML models were optimized for the total cohort. Interestingly, we expected that GBS and pre-endoscopic Rockall scores perform better in the upper GI subgroup, but similar AUC values and metrics were found as in the total cohort. The AUC of the ABC score was 0.77 for the total cohort and 0.76 for the upper GI subgroup.

Risk assessment of GIB can be key to identifying high-mortality risk patients so healthcare specialists can provide a more accurate and individualized healthcare service, increasing the probability of patients’ survival and reducing hospitalization costs. The source of bleeding can be identified with certainty in most cases during endoscopy, which can be 12–24 h later than the first meeting with the patient. Therefore, we recommend using risk assessment tools, which can equally be applied in non-variceal upper, variceal, or lower GI bleeding. Many ML risk assessment implements were configured only for upper or lower GIB patients, as listed in the systematic review of Shung et al.^1^.

Another noteworthy feature of our study is that with SHAP values, we created an opportunity to quickly visualize and easily explain our model’s risk stratification of individual patients. Users can simply understand the contributing features and their importance to a patient’s untoward outcome.

The Deshmukh et al.^20^. study focused on mortality risk assessment of critically ill GIB patients. They developed an ML model with a specificity of 27% and an AUC of 0.85, whereas the APACHE IVa clinical score had a specificity of 4% and an AUC of 0.80^20^. They adjusted the thresholds for both their ML model and the APACHE IVa score to achieve 100% sensitivity. In our study we did not make such adjustment to reach 100% sensitivity. Similarly to our study, they used the SHAP method to explain their prediction and ranked the top 25 clinical features contributing to their model. The first five features were: mean arterial pressure, bicarbonate, creatinine, polymorphonuclear leukocyte, heart rate, Glasgow Coma Scale^20^.

Our XGBoost and CatBoost model identified the patient’s CRP level as the most powerful characteristic influencing mortality. According to our knowledge, CRP was not involved in other previous GIB mortality risk prediction models. There are several publications^21–23^about routine blood tests, including CRP, that have a good predictive value among emergency department patients assessing short-term mortality. An interesting observation is that already hospitalized status (in-patients) contributes to higher mortality risk according to our ML models, which agrees with the results of the French cohort study^3^. Previous GIB episodes appear to be a protective factor; these patients can have a faster track in bleeding management or an earlier endoscopy, leading to lower mortality risk.

We compared the performances of our ML-based models to GBS, pre-endoscopic Rockall score and ABC score because these are the most widely used and studied conventional risk assessment tools.

In a retrospective study, Li et al. found that among six pre-endoscopic conventional scoring systems, ABC had the highest AUCs for the older and younger groups for predicting mortality (0.827 and 0.958, respectively)^15^. ABC score was calculated in 473 patients in our study, and the AUC 0.77 of this score is slightly lower than the performance of our ML-models, the sensitivity was 0.58. Due to our knowledge, our study is the first, where ABC risk score was compared with ML-based risk assessment tool in GI bleeding.

One of the first artificial neural network (ANN)-based models assessing the mortality risk of non-variceal upper GI patients was developed by Rotondano et al.^24^ In their study, 2,380 patients were involved, altogether 17 pre-endoscopic input variables were selected and used by the ANN, and the AUC was 0.95 with high sensitivity and specificity (83.8% and 97.5%). This model did not show the ranking due to the influence of the individual features contributing to the risk assessment. We also find it hard to calculate the time from symptoms to hospital admission because, in many cases, the patients cannot recall the first presentation of the GIB accurately, and the patients already in the hospital cannot be assessed with this model.

Shung et al.^25^ developed multiple ML models outperforming GBS, AIMS65, and pre-endoscopic Rockall scores in assessing a composite endpoint (mortality and interventions). This study’s strengths are the large, prospective cohort and their model has both external and internal validation. They used high sensitivity cut-off values (100%) to minimize false negative cases, and with this adjustment, the specificity was 26% of the best-performing ML model. With low specificity, there is high possibility of negative patients to treated like patients with mortality risk, which is not cost-effective.

In a similarly intriguing study of Shung et al.^26^ a recurrent neural network-based model was developed to dynamically predict need for packed red blood cell transfusion in the first 24 h of intensive care unit treatment of high-risk GI bleeding patients. Their model had an AUC of 0.81 in the internal validation set and 0.65 in the external validation set.

The main limitation of our study is that it lacks external validation, and the number of patients involved was moderate compared to other ML models. Data collection for the electronic GIB registry from two hospitals has the opportunity of human error during data input. Part of the registry’s data was retrospectively collected for consecutive patient involvement. We plan to make external validation of the developed ML risk assessment tool, and it is possible to analyze its performance, predicting other clinical outcomes such as rebleeding or need for intervention.

## Conclusion

Our study highlights that the new ML implementation has a good performance (AUC:0.84) in predicting in-hospital mortality of acute GIB patients, whereas the implementation of GBS and pre-endoscopic Rockall scores was rather poor. Using CatBoost, we reached a sensitivity of 78% and a specificity of 74%. Our newly developed ML models are useable in risk assessment for both upper and lower GI bleeding patients. Admission CRP level unexpectedly impacted in-hospital mortality outcomes.

## Methods

### Preliminary settings

Ethical permission for the study was given by the Scientific and Research Ethics Committee of the Hungarian Medical Research Council (24433-5/2019/EÜIG) in 2019, and we developed a uniform electronic clinical data registry for acute GIB patients. The study was conducted according to the Declaration of Helsinki, written informed consent was obtained from the participants. We prospectively and retrospectively collected data from patients who developed overt GIB between October 2019 and September 2022 in Pécs and between July 2021 and September 2022 in Székesfehérvár, Hungary.

Inclusion criteria were: age ≥ 18 years; GI bleeding at presentation or during any hospitalization manifested by melaena and/or haematochezia and/or haematemesis; and/or coffee-ground vomiting and/or verifiable drop of haemoglobin level. Patients with obscure GIB were excluded from the study.

Our observational cohort study is following the criteria of the Strengthening the Reporting of Observational Studies in Epidemiology (STROBE) reporting guidelines^27^. (Supplementary Table 2)

### Data collection

We recorded patient characteristics (age, sex, alcohol consumption, smoking, clinical signs of GIB); co-morbidities (hypertension, diabetes, cardiac disease, liver disease, chronic renal disease, malignancy, previous history of GIB); medication history (low-dose aspirin, clopidogrel, nonsteroidal anti-inflammatory drugs, anticoagulants, steroids); haemodynamic and other vital parameters (blood pressure, pulse rate, respiratory rate, oxygen saturation, Glasgow Coma Scale (GCS); laboratory results at presentation and during management; timing of endoscopy; findings at endoscopy; interventions during endoscopy; interventions during hospital care (need for ICU, surgery, transfusions); development of rebleeding; in-hospital mortality. We calculated GBS^6^and pre-endoscopic Rockall scores^7^and ABC scores^13^ of the included patients from our collected data.

During data collection, we grouped the GIB cases into five groups: nonvariceal UGIB, variceal UGIB, LGIB, small bowel bleeding, and iatrogenic. Iatrogenic bleeding was defined as GIB that occurred immediately after an endoscopic intervention or within 7–10 days.

### Data management

We applied a four-step data quality control system: after local administrative validation and local medical approval, the study coordination team undertook a central registry administrative and an expert gastroenterologist’s check. Then, with an expert statistician, the study team validated and checked the missing data and the outliers of the raw data set.

### Developing a risk assessment tool with machine learning models

Our goal was to develop an ML model that predicts the in-hospital mortality risk of GIB patients.

First, every categorical information was converted into numerical variables with a one-hot encoding method. First, the variables where missing values reached 30% were excluded from the analysis. To handle remaining missing data, we used the IterativeImputer approach^28^. Since death occurred in 11% of the cases, our dataset was considered imbalanced. To overcome the imbalance between severe and not severe cases, we applied the Synthetic Minority Oversampling Technique (SMOTE) to oversample severe cases. With StratifiedKFold we performed internal validation using 5-fold cross-validation, which means splitting the dataset into 5 equal parts (folds), training the model on 4 of the folds, and validating on the remaining fold, repeating 5 times a different fold as the validation set and averaging the performance metrics obtained from each fold. For the modeling, we used XGBoost^29^and CatBoost^30^ algorithms, both decision tree models using extreme gradient boosting.

We used forward selection of variables according to their Predictive Power Score (PPS), and we selected the variables to get the highest AUC values. Forward selection is a step-by-step process where variables are added one by one based on their predictive strength, stopping when model performance no longer improves. We applied hyperparameter optimization on both XGBoost and CatBoost models, which had the variables with the best PPS. To evaluate our two models, we compared the area under the receiver operating characteristic curve (AUC) with its 95% confidence interval (CI) and other metrics (sensitivity, specificity, F1 score, accuracy, and precision) of the models. Sensitivity shows how good the model is in finding true positive (death) cases. F1 score combines precision and recall. After a long variable selection process, we identified the final 19 variables to train and cross-validate our models.

AUCs of the developed machine learning predictive models and the GBS, pre-endoscopic Rockall and ABC scores were compared. The cut-off values of GBS, Rockall-score and ABC score were 11, 4 and 8, respectively, to identify high-risk cases according to previous studies^3^.

We measured the performance and metrics of the two ML-based models and the conventional risk assessment tools also in the subgroup of upper GI bleeding patients.

### Interpretation of the risk assessment model

We worked with the SHapley Additive exPlanations (SHAP) tool^31^ to explain the most critical variables and their contribution to the mortality risk assessment. The Shapley value quantifies the contribution of each variable to the final prediction of individual patients. SHAP helps in understanding the feature’s importance on the whole cohort globally and provides insight into how the features influence the model’s output for an individual patient.

### Statistical analyses

Case numbers and percentages were calculated for categorical variables, and mean with standard deviation (SD) and median with interquartile range (IQR) were calculated for numerical variables in descriptive analyses of the original cohort. A two-sided p-value of < 0.05 was considered statistically significant.

## Electronic supplementary material

Below is the link to the electronic supplementary material.

Supplementary Material 1

## Data Availability

The datasets generated and analysed during the current study are not publicly available in The Hungarian Gastrointestinal Bleeding Registry but are available from the corresponding author on reasonable request.
