# Supplementary material for: New machine-learning models outperform conventional risk assessment tools in Gastrointestinal bleeding
Source: Sci Rep. 2025 Feb 21;15:6371. doi: 10.1038/s41598-025-90986-1 (PMC11845789; doi:10.1038/s41598-025-90986-1)
Supplement: Supplementary file 1 — Supplementary Material 1 [file 41598_2025_90986_MOESM1_ESM.docx]

**Supplementary Material**

New machine-learning models outperform conventional risk assessment tools in gastrointestinal bleeding.

Eszter Boros ^1,5^, József Pintér^2^, Roland Molontay^2,7^, Kristóf Gergely Prószéky^2^, Nóra Vörhendi^1,9^, Orsolya Anna Simon ^1,6^, Brigitta Teutsch ^1,3^ Dániel Pálinkás ^3,10^, Levente Frim^1^, Edina Tari^3,4^, Endre Botond Gagyi^3,11^, Imre Szabó^6^, Roland Hágendorn^6^, Áron Vincze^6^, Ferenc Izbéki^5^, Zsolt Abonyi-Tóth^3^, Andrea Szentesi^1^, Vivien Vass^1^, Péter Hegyi ^1,3^, Bálint Erőss ^1,3,⃰^

^1^Institute for Translational Medicine, Medical School, University of Pécs, Pécs, Hungary

^2^ Department of Stochastics, Institute of Mathematics, Budapest University of Technology and Economics, Budapest, Hungary

^3^Centre for Translational Medicine, Semmelweis University, Budapest, Hungary

^4^Institute of Pancreatic Diseases, Semmelweis University, Budapest, Hungary

^5^Fejér County Szent György University Teaching Hospital, Székesfehérvár, Hungary

^6^ First Department of Medicine, Medical School, University of Pécs, Pécs, Hungary

^7^ Institute of Biostatistics and Network Science, Semmelweis University, Budapest, Hungary

^8^Department of Biostatistics, University of Veterinary Medicine, Budapest, Hungary

^9^ Internal Medicine, Hospital and Clinics of Siófok, Siófok, Hungary

^10^Department of Gastroenterology, Central Hospital of Northern Pest – Military Hospital, Budapest, Hungary

^11^Selye János Doctoral College for Advanced Studies, Semmelweis University, Budapest, Hungary

⃰Corresponding author

**Supplementary Table 1.** Basic metrics of the Risk Assessment Tools

|  | **SET** | **AUC** | **Specificity** | **Sensitivity** | **Accuracy** | **Precision** | **F1-score** |
| --- | --- | --- | --- | --- | --- | --- | --- |
| **XGBoost** | **FULL** | 0.84 (0.76-0.90) | 0.96 (0.92-0.98) | 0.25 (0.10-0.43) | 0.88 (0.85-0.91) | 0.40 (0.17-0.64) | 0.30 (0.12-0.47) |
| **CatBoost** | **FULL** | 0.84 (0.77-0.90) | 0.74 (0.66-0.83) | 0.78 (0.57-0.95) | 0.75 (0.69-0.80) | 0.26 (0.21-0.33) | 0.39 (0.31-0.47) |
| **XGBoost** | **Upper GI** | 0.79 (0.72-0.86) | 0.94 (0.89-0.98) | 0.27 (0.12 - 0.43) | 0.85 (0.8-0.89) | 0.42 (0.23-0.72) | 0.32 (0.15-0.47) |
| **CatBoost** | **Upper GI** | 0.79 (0.71-0.88) | 0.63 (0.51-0.71) | 0.79 (0.58-0.99) | 0.66 (0.56-0.72) | 0.26 (0.21-0.33) | 0.39 (0.32-0.44) |
| **ABC** | **FULL** | 0.77 (0.71 - 0.83) | 0.80 (0.76 - 0.84) | 0.58 (0.43 - 0.73) | 0.77 (0.74 - 0.81) | 0.28 (0.21 - 0.37) | 0.38 (0.29 - 0.47) |
| **GBS** | **FULL** | 0.68 (0.62 - 0.74) | 0.68 (0.65 - 0.71) | 0.61 (0.51 - 0.71) | 0.67 (0.64 - 0.70) | 0.17 (0.13 - 0.21) | 0.27 (0.21 - 0.33) |
| **Rockall** | **FULL** | 0.62 (0.56 - 0.67) | 0.64 (0.6 - 0.67) | 0.52 (0.43 - 0.62) | 0.62 (0.59 - 0.65) | 0.14 (0.11 - 0.18) | 0.22 (0.17 - 0.27) |
| **ABC** | **Upper GI** | 0.76 (0.70 - 0.83) | 0.78 (0.74 - 0.83) | 0.63 (0.49 - 0.77) | 0.76 (0.72 - 0.80) | 0.34 (0.27 - 0.43) | 0.44 (0.35 - 0.53) |
| **GBS** | **Upper GI** | 0.62 (0.56 - 0.70) | 0.56 (0.52 - 0.60) | 0.65 (0.57 - 0.75) | 0.57 (0.54 - 0.61) | 0.18 (0.14 - 0.24) | 0.28 (0.23 - 0.35) |
| **Rockall** | **Upper GI** | 0.61 (0.55 - 0.67) | 0.62 (0.58 - 0.66) | 0.54 (0.42 - 0.64) | 0.61 (0.57 - 0.64) | 0.18 (0.13 - 0.22) | 0.27 (0.21 - 0.33) |

*Mean values are represented with 95% confidence intervals.*

**Supplementary Table 2:** STROBE Statement—checklist of items that should be included in reports of observational studies

|  | | **Item No.** | **Recommendation** | **Page  No.** | | **Relevant text from manuscript** |
| --- | --- | --- | --- | --- | --- | --- |
| **Title and abstract** | | 1 | (*a*) Indicate the study’s design with a commonly used term in the title or the abstract | 2 | | We analyzed the prospective, multicenter Hungarian GIB Registry's data. |
|  |  |  | (*b*) Provide in the abstract an informative and balanced summary of what was done and what was found | 2 | |  |
| **Introduction** | | | | | |  |
| Background/rationale | | 2 | Explain the scientific background and rationale for the investigation being reported | 3 | |  |
| Objectives | | 3 | State specific objectives, including any prespecified hypotheses | 4 | | In this study, we aimed to develop and validate ML models to calculate the risk of in-hospital mortality in patients admitted for overt GIB |
| **Methods** | | | | | |  |
| Study design | | 4 | Present key elements of study design early in the paper | 11 | |  |
| Setting | | 5 | Describe the setting, locations, and relevant dates, including periods of recruitment, exposure, follow-up, and data collection | 11 | |  |
| Participants | | 6 | (*a*) *Cohort study*—Give the eligibility criteria, and the sources and methods of selection of participants. Describe methods of follow-up  *Case-control study*—Give the eligibility criteria, and the sources and methods of case ascertainment and control selection. Give the rationale for the choice of cases and controls  *Cross-sectional study*—Give the eligibility criteria, and the sources and methods of selection of participants | 11 | |  |
|  |  |  | (*b*) *Cohort study*—For matched studies, give matching criteria and number of exposed and unexposed  *Case-control study*—For matched studies, give matching criteria and the number of controls per case |  | |  |
| Variables | | 7 | Clearly define all outcomes, exposures, predictors, potential confounders, and effect modifiers. Give diagnostic criteria, if applicable | 11-12 | |  |
| Data sources/ measurement | | 8* | For each variable of interest, give sources of data and details of methods of assessment (measurement). Describe comparability of assessment methods if there is more than one group | *12* | |  |
| Bias | | 9 | Describe any efforts to address potential sources of bias | 12 | |  |
| Study size | | 10 | Explain how the study size was arrived at | 11 | |  |
| Quantitative variables | 11 | | Explain how quantitative variables were handled in the analyses. If applicable, describe which groupings were chosen and why | 12 |  | |
| Statistical methods | 12 | | (*a*) Describe all statistical methods, including those used to control for confounding | 12-13-14 |  | |
|  |  |  | (*b*) Describe any methods used to examine subgroups and interactions | 13 |  | |
|  |  |  | (*c*) Explain how missing data were addressed | 12 | First, the variables where missing values reached 30% were excluded from the analysis. | |
|  |  |  | (*d*) *Cohort study*—If applicable, explain how loss to follow-up was addressed  *Case-control study*—If applicable, explain how matching of cases and controls was addressed  *Cross-sectional study*—If applicable, describe analytical methods taking account of sampling strategy |  | Not applicable. | |
|  |  |  | (*e*) Describe any sensitivity analyses |  | Not applicable. | |
| **Results** | | | | | | |
| Participants | 13* | | (a) Report numbers of individuals at each stage of study—eg numbers potentially eligible, examined for eligibility, confirmed eligible, included in the study, completing follow-up, and analysed | 4 |  | |
|  |  |  | (b) Give reasons for non-participation at each stage | 4 |  | |
|  |  |  | (c) Consider use of a flow diagram | Not applicable. |  | |
| Descriptive data | 14* | | (a) Give characteristics of study participants (eg demographic, clinical, social) and information on exposures and potential confounders | 4 |  | |
|  |  |  | (b) Indicate number of participants with missing data for each variable of interest | 4 | Table 1 | |
|  |  |  | (c) *Cohort study*—Summarise follow-up time (eg, average and total amount) | Not applicable. |  | |
| Outcome data | 15* | | *Cohort study*—Report numbers of outcome events or summary measures over time | 4-5-6 |  | |
|  |  |  | *Case-control study—*Report numbers in each exposure category, or summary measures of exposure |  |  | |
|  |  |  | *Cross-sectional study—*Report numbers of outcome events or summary measures |  |  | |
| Main results | 16 | | (*a*) Give unadjusted estimates and, if applicable, confounder-adjusted estimates and their precision (eg, 95% confidence interval). Make clear which confounders were adjusted for and why they were included | 4-5-6 |  | |
|  |  |  | (*b*) Report category boundaries when continuous variables were categorized | 4-5-6 |  | |
|  |  |  | (*c*) If relevant, consider translating estimates of relative risk into absolute risk for a meaningful time period | Not applicable. |  | |

| Other analyses | 17 | Report other analyses done—eg analyses of subgroups and interactions, and sensitivity analyses | 5 |  |
| --- | --- | --- | --- | --- |
| **Discussion** | | | | |
| Key results | 18 | Summarise key results with reference to study objectives | 6-7-8 |  |
| Limitations | 19 | Discuss limitations of the study, taking into account sources of potential bias or imprecision. Discuss both direction and magnitude of any potential bias | 9-10 |  |
| Interpretation | 20 | Give a cautious overall interpretation of results considering objectives, limitations, multiplicity of analyses, results from similar studies, and other relevant evidence | 9-10 |  |
| Generalisability | 21 | Discuss the generalisability (external validity) of the study results | 10-11 |  |
| **Other information** | |  | | |
| Funding | 22 | Give the source of funding and the role of the funders for the present study and, if applicable, for the original study on which the present article is based | 18 |  |


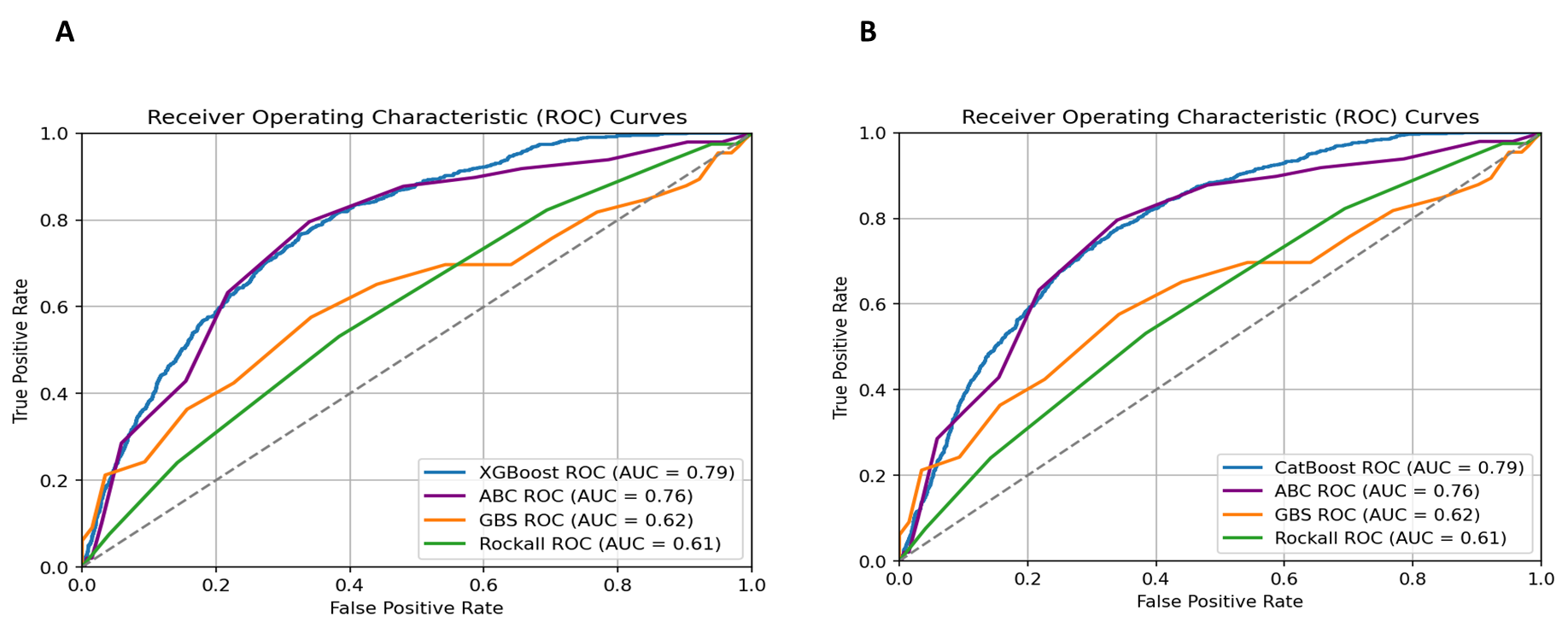


**Supplementary Figure 1: ROC curves of the machine-learning models compared with the performance of Glasgow-Blatchford, pre-endoscopic Rockall and ABC scoring systems calculated in the upper GI bleeding subgroup.**

Figure 1A represents the XGBoost model. Figure 1B represents CatBoost model. AUC: area under the receiver operating characteristic curve, GBS: Glasgow-Blatchford score, ROC: receiver operating characteristic
